# Sepsis and AKI in Clinical Emergency Room Patients: The Role of Urinary NGAL

**DOI:** 10.1155/2015/413751

**Published:** 2015-07-14

**Authors:** Hong Si Nga, Pamela Medeiros, Precil Menezes, Ramaiane Bridi, André Balbi, Daniela Ponce

**Affiliations:** São Paulo State University (UNESP), Distrito de Rubião Junior, s/n, 18618-970 Botucatu, SP, Brazil

## Abstract

*Background*. Few studies have investigated the predictive properties of urinary (u) NGAL as an AKI marker in septic population. *Objectives*. This study evaluated the efficacy of uNGAL as predictor of AKI and death in septic patients admitted to the clinical emergency room (ER). *Methodology*. We prospectively studied patients with sepsis admitted to the ER. Urine was analyzed for NGAL within the first 24 hours after admission (classified as NGAL1), between 24 and 48 h (NGAL2), and at moment of AKI diagnosis (NGAL3). *Results*. Among 168 septic patients admitted to ER, 72% developed AKI. The uNGAL and its relationship with creatinine (Cr) were high in septic patients but statistically higher in those with sepsis and AKI. The uNGAL1 and uNGAL2, as well as uNGAL1/uCr1 and uNGAL2/uCr2, were good predictors for AKI (AUC-ROC 0.73, 0.70, 0.77, and 0.84, resp.). The uNGAL1 and uNGAL1/uCr1 were poor predictors for death (AUC-ROC 0.66 and 0.68, resp.), whereas uNGAL2 and uNGAL2/uCr2 were better predictors (AUC-ROC 0.70 and 0.81, resp.). *Conclusion*. The uNGAL is highly sensitive but nonspecific predictor of AKI and death in septic patients admitted into ER.

## 1. Introduction

Sepsis is defined as systemic inflammatory response syndrome associated with infection. It is a primary cause of morbidity and mortality in patients admitted to emergency clinical room (ER) and in intensive care units (ICU) [1–3]. It is a well-known risk factor for the development of acute kidney injury (AKI), occurring in approximately 51% of patients with septic shock [4–6]. The presence of AKI leads to significant impact on morbidity, increased length of stay in hospital, and high costs, and it is an independent risk factor for mortality [4, 7, 8].

Neutrophil gelatinase-associated lipocalin (NGAL) is a rising biomarker for early diagnosis of AKI in different scenarios. NGAL levels in both plasma (p) and urine (u) increase soon after the renal insult and they seem to detect AKI hours or days before creatinine (Cr) [9–12]. Although considered an early biomarker, NGAL levels can be elevated after activation of neutrophils, suggesting influence of systemic inflammation and infections [13–16].

Few studies have investigated the predictive properties of NGAL as an AKI marker in a septic population. Studies on pediatric ICU patients have shown pNGAL to be a nonspecific predictor [17] and uNGAL to be a good predictor of AKI [18]. In these two pediatric studies AKI and sepsis coincided to a great extent. This is common in ICU patients and might obstruct the interpretation of elevated NGAL in plasma and urine. Indeed, Bagshaw et al. [19] in a study that included 83 AKI patients showed that both p- and uNGAL were higher in septic versus nonseptic patients. Mårtensson et al. [20] performed a study that evaluated 65 septic patients admitted to ICU and showed that pNGAL was not a good predictor of AKI because it was elevated in septic patients without AKI, probably due to the systemic infections.

Given the higher mortality rate of patients with sepsis and AKI and lack of studies in ER, we decided to investigate the role of uNGAL as predictor of AKI and death in septic patients admitted to ER.We believe uNGAL is predictor of AKI and death in septic patients admitted to ER.

## 2. Materials and Methods

### 2.1. Study Population

**Figure 1:**
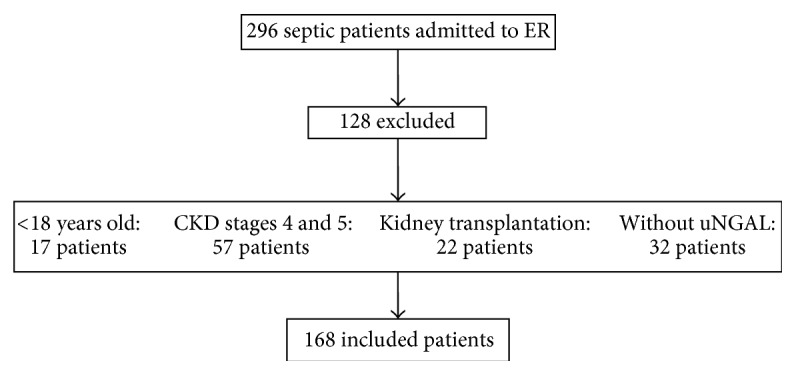
Screening and enrollment.

We screened all septic patients admitted through the internal medicine department to the ER of our University Hospital from January 2013 to May 2014 for enrollment in a prospective cohort study designed to study the development of AKI following sepsis. We included patients 18 years of age or older who had sepsis according to “Survival Sepsis Campaign 2012” [21] and exclusion criteria were patients with chronic kidney disease stages 4 and 5 (creatinine clearance lower than 30 mL/min/1.73 m^2^) estimated by the modification of diet in renal disease (MDRD) equation [22] and patients undergoing kidney transplantation. Complete data on inclusions and exclusions are shown in Figure 1.

The Ethics Committee of the Botucatu School of Medicine, UNESP, approved this study with a waiver of informed consent given its observational nature.

AKI was defined and classified according to AKIN criteria [23, 24]. Baseline Cr was defined as the lowest Cr value in the last 6 months before AKI or, for those without this measurement, the lowest value achieved during hospitalization in the absence of dialysis [25, 26].

AKI was considered to have occurred on the first day that any criterion was met, though full staging continued through discharge from hospital or death, whichever came first. Day 0 was defined as the calendar day of ER, and thus its length varied depending on time of presentation. We determined vital status at the time of discharge from the hospital for all patients.

Our 5-day study time frame was designed to capture most cases of early AKI and to reduce the confounding effects of time-varying interventions (e.g., nephrotoxic medications) and late complications (e.g., sepsis related to mechanic ventilation (MV)) which would likely have had a greater impact in cases of AKI occurring later in the hospital course.

### 2.2. Biochemical Analysis

Samples of blood were collected once daily during 5 days or earlier if discharged from hospital or death. Urine was analyzed for NGAL and Cr within the first 24 hours after admission (classified as NGAL1), between 24 and 48 h (NGAL2), and at moment of AKI diagnosis (NGAL3). The samples were centrifuged and stored at minus 80-degree Celsius and were analyzed subsequently. NGAL was measured by the* enzyme linked immunosorbent assay* (ELISA).

Expected normal uNGAL level was less than 0.2 ng/mL. We performed dosage of uNGAL in 20 healthy subjects between 30 and 50 years old and the mean was 0.2 ± 0.029 ng/mL.

### 2.3. Statistical Analysis

Data analysis was performed using SAS for Windows (version 9.2, SAS Institute, Cary, NC, USA, 2012). Continuous variables with normal distribution were described using means ± standard deviation and those with a nonnormal distribution as median and interquartile range. Categorical variables were presented as *n* (%). For the analysis of continuous variables, Student's *t*-test was used for data with a parametric distribution and the Kruskal-Wallis test for nonnormal data. For the analysis of categorical variables a chi-square test was used.

Diagnostic characteristics of uNGAL in predicting AKI and death were assessed by calculation of the area under the receiver operating characteristic curve (AUC-ROC). AUC-ROC analysis was performed by comparing AKI patients with all non-AKI patients and by comparing survivor patients with those nonsurvivor patients. In all tests, differences were considered significant at 5%.

## 3. Results

One hundred sixty-eight patients were included in the final analysis (Figure 1). Mean age was 68.0 ± 15.4 years, 57.7% were male, most of them had comorbidities (65.4%), and hypertension, cardiovascular disease, and diabetes mellitus were the most frequent (in 50.6, 29.7, and 26.1% of patients, resp.). APACHE II score was 19.70 ± 7.1 and the need for mechanical ventilation and noradrenalin use in the first 24 hours after admission to ER was 21.4 and 55.4%, respectively. Septic shock was the primary sort of septic state (55.4%). The main source of infection was the lung (48.8%), followed by the urinary tract (18%). The pathogen was isolated in culture in only 38% of cases. Patients were mainly directed towards the ICU (51.8%), while only 53 patients (31.5%) were referred to the ward and the others stayed at the ER. Within the first five ER days, 121 subjects (72%) developed AKI. The mortality rate was 44% (Table 1).

Among AKI patients, 87 (71.9%) already had the diagnosis on admission to the ER, while 34 of them developed AKI during hospitalization. Most of patients were classified as AKIN 3 (43%), while AKIN 1 occurred in 35 patients (28.9%) and AKIN 2 in 34 (28.1%).

uNGAL1 and uNGAL2 in AKI group showed higher values than non-AKI group: 3.86 (2.6 to 9.5) versus 3.5 (0.8–5); *p* = 0.003 and 3.03 (0.65–4.33) versus 2.76 ng/mL (2.3–7.83); *p* = 0.009, respectively, and uNGAL/uCr in the first 24 h: 75.08 (37–165) × 53.31 (17.79 to 102.2); *p* < 0.0001, and between 24 and 48 h after admission: 77.2 (29.4 to 160.6) × 60.29 (17.56 to 85.64); *p* = 0.02 (Table 2).

uNGAL1 was higher in nonsurvival group when compared with survival patients (4.88 (2.19–9.51) × 3.30 ng/mL (1.76–6.18), *p* = 0.01). The two groups were similar in uNGAL2 (*p* = 0.16), as well as uNGAL/uCr in the first 24 h (*p* = 0.72) and between 24 and 48 h after admission (*p* = 0.63) (Table 3).

**Figure 2:**
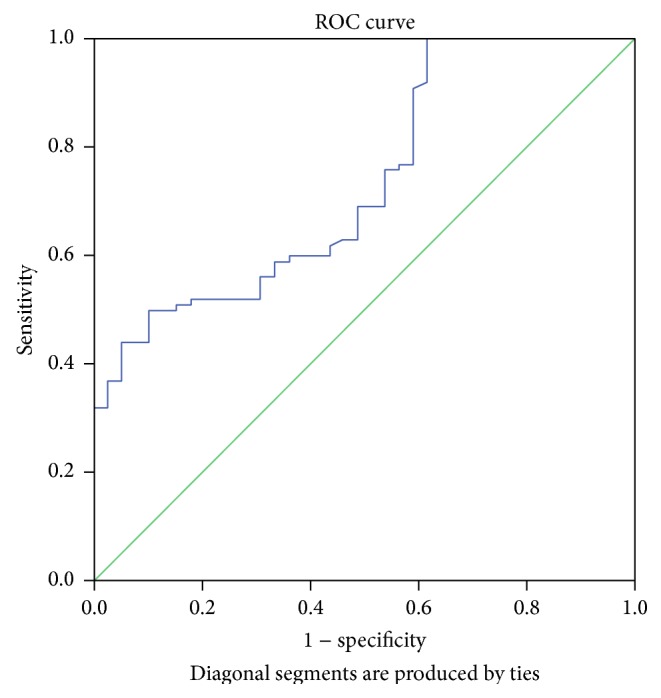
ROC analysis of uNGAL measure on day 1 of admission to the ER in septic patients with AKI versus without AKI.

**Figure 3 fig3:**
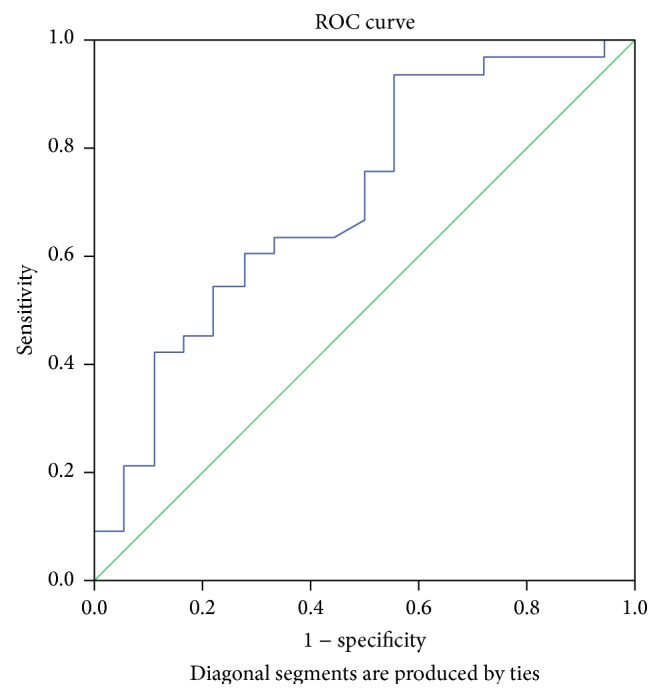
ROC analysis of uNGAL measure on day 2 of admission to the ER in septic patients with AKI versus without AKI.

**Figure 4 fig4:**
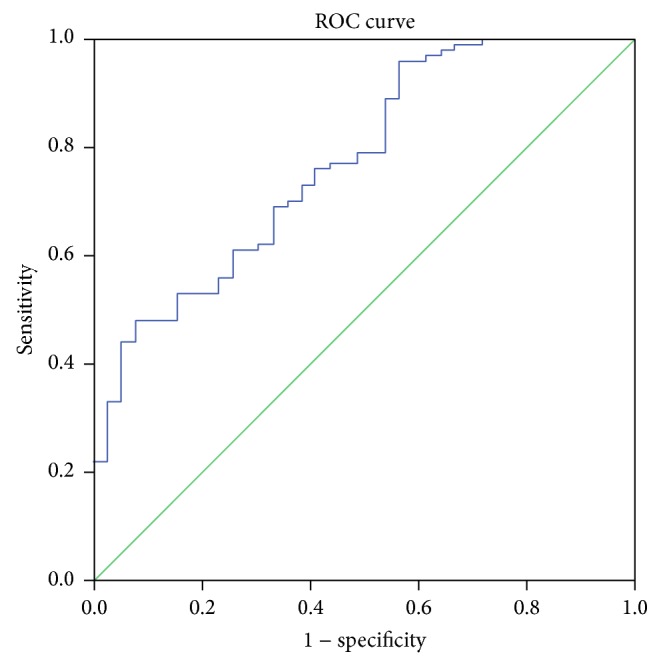
ROC analysis of uNGAL/uCr measure on day 1 of admission to the ER in septic patients with AKI versus without AKI.

**Figure 5:**
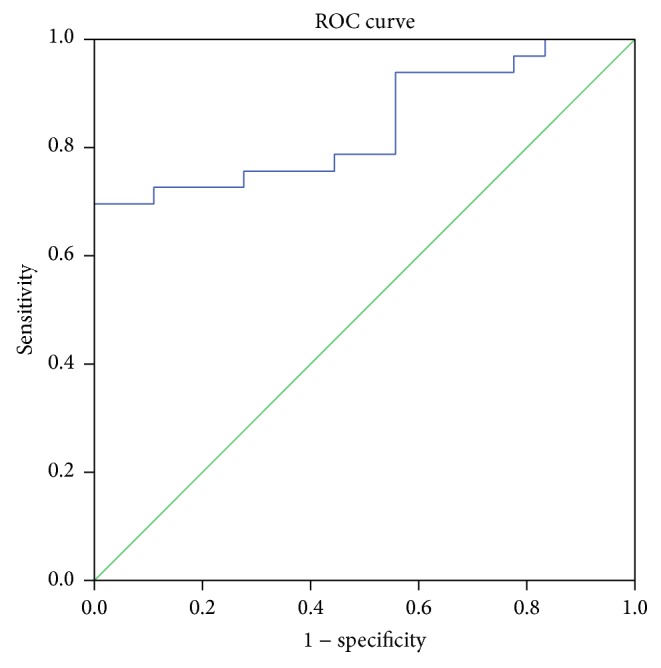
ROC analysis of uNGAL/uCr measure on day 2 of admission to the ER in septic patients with AKI versus without AKI.

Figures 2 [Fig fig3] [Fig fig4]–5 display the receiver operator curves (ROC) for uNGAL as predictor of AKI. The areas under the curve for uNGAL1, uNGAL2, uNGAL1/uCr1, and uNGAL2/uCr2 were 0.73, 0.70, 0.77, and 0.84, respectively. Both uNGAL and uNGAL/uCr were good predictors of AKI within the next 48 h. The optimal cutoff value of each one of them had sensitivity and specificity of 0.63 and 0.46, 0.63 and 0.44, 0.7 and 0.38, and 0.75 and 0.43, respectively (Table 4).

**Figure 6:**
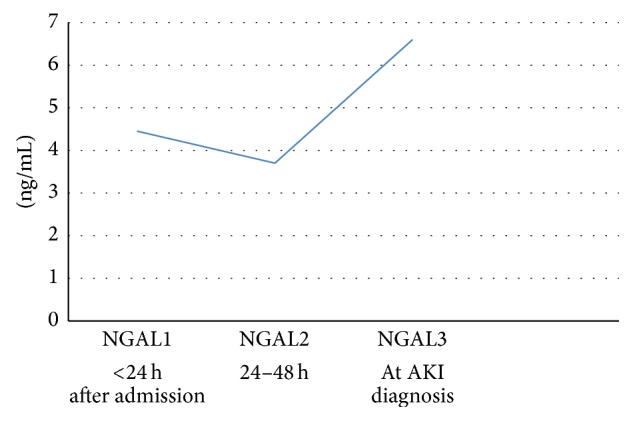
Urinary NGAL values at three moments after admission in septic patients undergoing AKI during hospitalization.

Figure 6 shows the values of uNGAL at different moments (1, 2, and 3) in the group of septic patients that developed AKI during the hospitalization. The expression of uNGAL seemed to follow a bimodal pattern around the development of AKI with an early peak preceding AKI followed by a second peak after AKI was established, which was observed only in patients with no AKI at admission.

Subanalysis was performed involving only patients who did not present AKI at admission to ER (*n* = 81) and results were better than those described in the general population (*n* = 168 patients). The areas under the curve for uNGAL1, uNGAL2, uNGAL1/uCr1, and uNGAL2/uCr2 were 0.83, 0.81, 0.87, and 0.89, respectively. Both uNGAL and uNGAL/uCr were excellent predictors of AKI within the next 48 h. The optimal cutoff value of each one of them had sensitivity and specificity of 0.77 and 0.66, 0.75 and 0.78, 0.81 and 0.68, and 0.79 and 0.67, respectively (Table 5).

**Figure 7:**
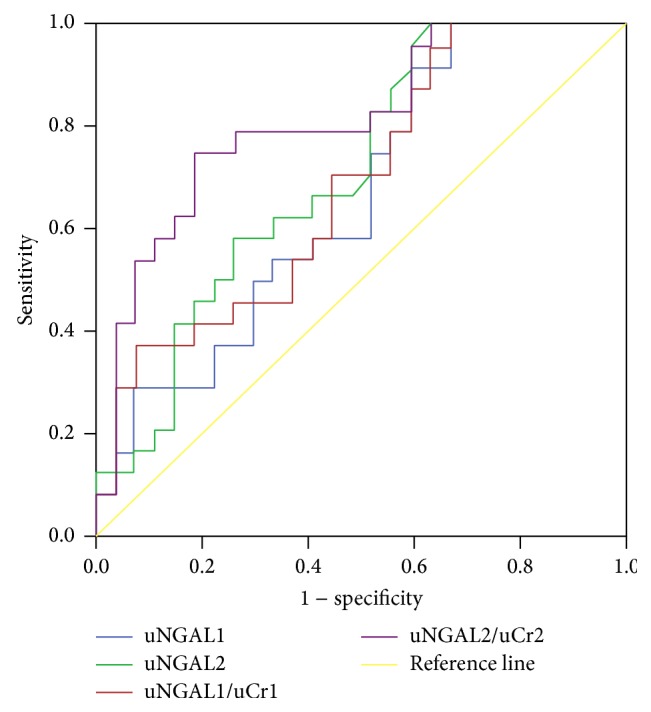
ROC analysis of uNGAL and uNGAL/uCr measured on days 1 and 2 of the admission to ER in survivors versus nonsurvivors septic patients.

Concerning uNGAL as predictor of death, the areas under the curve for uNGAL1, uNGAL2, uNGAL1/uCr1, and uNGAL2/uCr2 were 0.66, 0.70, 0.68, and 0.81, respectively (Figure 7). Only uNGAL2 and uNGAL2/uCr2 were good predictors of septic patients death. The optimal cutoff value of each one of them had sensibility and specificity of 0.88 and 0.54, 0.95 and 0.59, 0.71 and 0.45, and 0.71 and 0.48, respectively (Table 6).

## 4. Discussion

This is the first study of septic adult patients admitted to ER to undergo prospective evaluation of uNGAL as a biomarker for AKI and death. Among 168 patients with sepsis and septic shock admitted into ER, 121 (72%) developed AKI defined by AKIN classification [24] and mortality rate was 44%. There are few studies on AKI in ER and these findings are consistent with the previous studies performed in ICU.

Many studies have reported that AKI is more frequently observed in patients with sepsis and septic shock than in patients with other conditions [23, 24, 27, 28]. An observational cohort study of 390 patients with septic shock in a single center ICU for about 2 years reported nearly 2 out of 3 patients experiencing AKI. In a recent retrospective multicenter study of 4532 patients with septic shock, a similar percentage of patients (64.4%) developed AKI [27]. Challiner et al. [28] performed a retrospective study with 745 patients admitted to the emergency department and evaluated the presence or absence of AKI according to AKIN criteria. AKI incidence was 25.4% overall, with approximately one-third present on admission and two-thirds developing after admission.

Herein we show that uNGAL and its relation to uCr were significantly increased within the first 48 hours of admission to the ER in septic AKI patients compared to healthy controls and septic patients without AKI. In addition, uNGAL on day 1 of admission to ER was significantly increased in nonsurvivors septic patients compared to survivors ones. uNGAL therefore appears to be a highly sensitive predictor of AKI and death in this population.

NGAL is a protein with a molecular weight of 25 kDa expressed at low concentrations in different tissues and upregulated especially in injured epithelial cells. Because of that, pNGAL concentration can be high in septic patients, even in the absence of AKI. Then, it may be considered a marker of sepsis, as well as an early biomarker of AKI [29], as shown in several clinical studies [9, 10, 18, 30–32].

In study of Dellinger et al. [21], pNGAL and uNGAL were evaluated as predictors of AKI, but the ability of pNGAL to predict AKI in patients with septic shock was poor with an AUC-ROC (0.67) compared to the ability of uNGAL with an AUC-ROC (0.86). The uNGAL was a better predictor of AKI in septic patients than pNGAL probably because it was less affected by presence of sepsis. The pNGAL can be high because of its release into the bloodstream by the systemic activation of neutrophils due to sepsis. The physiological function of the uNGAL is unknown; however, it has a role in renal morphogenesis [33]. The proteomic analysis of studies using animal models revealed uNGAL protein as the earliest product after kidney insult [31], representing better the kidney damage than the pNGAL.

Similar results were found in pediatric patients [17] also with septic shock in ICU and the AUC-ROC (0.67), shown to be more sensitive predictor than specific. As the proper sepsis activates and increases the release of NGAL from neutrophils, it is questionable whether it can impair the ability to predict AKI.

In the current study, uNGAL in healthy adults was much lower (median 0.2 ng/mL, IQR 0–1.1 ng/mL) than that reported in other studies [18–20]. These differences are likely related to the different techniques used to measure NGAL in different studies.

In our study both uNGAL and its relation to uCr on day 1 and day 2 after admission of septic patients to the ER were good predictors of AKI. In the current study, ROC analysis suggested that uNGAL2/uCr2 had an excellent accuracy (0.84) and a high sensitivity for predicting AKI (75%), albeit with relatively poor specificity (46%) predictor of AKI in septic patients admitted to ER. In subanalysis that involved only patients who did not present AKI at admission to ER (*n* = 81), the results were better than those described in the general population. ROC analysis showed that uNGAL and NGAL/uCr were excellent predictors of AKI within the next 48 h (>0.8), with a high sensitivity (>75%) and a good specificity (>65%).

Our results are similar to the AUC-ROC found in pediatric septic patients by Wheeler et al. [17] and in adults septic patients in study performed by Mårtensson et al. [20]. The authors showed that the AUC was 0.677 (95% CI 0.557, 0.786; *p* = 0.008) with an optimal cutoff value of 139 ng/mL (sensitivity = 86% and specificity = 39%).

However, in our study, uNGAL was measured within the first 48 hours of admission to the ER, which is not necessarily the first 48 hours of their disease process. In fact, the vast majority of these patients already had the sepsis diagnosis on admission to the ER. We would therefore expect the uNGAL concentrations to be much higher in patients with septic shock and evolving kidney injury.

In a study that followed children undergoing cardiopulmonary bypass and analyzed uNGAL and pNGAL as predictors of AKI, the concentration of uNGAL greater than 50 *μ*g/L predicted AKI at two hours following procedure in this population, with 100% sensibility and 98% specificity, while pNGAL concentrations greater than 25 *μ*g/L did not show such good results, with 70% sensibility and 94% specificity [9].

We also analyzed the values of uNGAL at different moments (1 and 2) in the 34 patients that developed AKI during the hospitalization. The expression of uNGAL seemed to follow a bimodal pattern around the development of AKI with an early peak preceding AKI followed by a second peak after AKI was established. Similar results were found by Mårtensson et al. [20]. The first peak was attributed to the excretion of NGAL from neutrophils sequestered in renal tubule and the second peak represented the expression of NGAL released by the tubular cells themselves. Cai et al. [34] studied patients who underwent cardiac surgery and found different molecular forms of uNGAL measured by ELISA at different time points.

We did not find the same for the group that already had AKI by admission; the explanation for that is the fact that the values of NGAL by admission represent the second peak and probably the biomarker should be elevated hours or even days before the hospitalization.

Concerning uNGAL as predictor of death, in our study uNGAL1 and uNGAL1/uCr1 were poor predictors (AUC-ROC was 0.7). The uNGAL2 and the uNGAL2/uCr2 were good predictor of death in septic patients. ROC analysis suggested that uNGAL2 had a good accuracy (0.7) and high sensitivity for predicting death (95%), whereas uNGAL2/uCr2 was better, with an excellent accuracy (0.81) and sensitivity for predicting death (71%), albeit nonspecific predictor of death (48%) in septic patients admitted to ER. We believe that adding any other marker, KIM-1, for example, with higher specificity, would help to improve the predictive value of the studied markers.

We speculate that uNGAL2 was better predictor of death in septic patients than uNGAL1 because it may reflect the patient's response to initial treatment of sepsis. If after 24 hours of initial treatment the NGAL u 2 and the uNGAL2/uCr2 remain high, they can predict death of septic patients admitted to ER.

Few studies have shown an association between NGAL and mortality. Nickolas et al. [35] showed that uNGAL was associated with clinical outcomes, including consultation with nephrologist, dialysis, and ICU admission (OR = 24.71 (CI: 7.69 to 79.42)). Collins et al. [36] evaluated 399 patients with acute cardiac dysfunction and found that uNGAL between 12 and 24 h after treatment initiation was predictive of 30-day mortality (*p* = 0.02).

The present study has some important limitations. It included a small number of patients and was performed in single center. Due to the small number of patients, no analysis of uNGAL according to the stage of AKI or classification of sepsis was performed. The role of uNGAL as a predictor of dialysis also was not evaluated. Despite these limitations, the results of this study allow us to conclude that uNGAL is elevated in septic patients but statistically higher in those with sepsis and AKI and reaffirm the role of uNGAL to predict AKI and death. uNGAL/uCr values on day 2 after admission to ER were the best predictors of AKI and death in septic patients, with being highly sensitive, but nonspecific. We speculate that uNGAL values may be confounded by hydration status and urine output and may therefore need standardization by expressing as a ratio with uCr.

The uNGAL is a highly sensitive but nonspecific predictor of AKI and death in septic patients admitted into ER and further validation of uNGAL as a biomarker of AKI in this population is warranted.

## Figures and Tables

**Table 1:** Patients demographics and clinical characteristics (*n* = 168).

Characteristics	Septic patients (*N* = 168)
Male sex *n* (%)	97 (57.7)
Age (years)	68 ± 15.4
MBP	73.3 ± 23
Comorbidities *n* (%)	
Hypertension	85 (50.6)
Diabetes	44 (26.1)
Dyslipidemia	18 (10.7)
Cardiovascular disease	50 (29.7)
Liver disease	6 (3.5)
CKD	12 (7.1)
Baseline creatinine	0.82 ± 0.3
Noradrenaline use *n* (%)	93 (55.3)
Classification of sepsis *n* (%):	
Sepsis	26 (15.4)
Severe sepsis	49 (29.1)
Septic shock	93 (55.3)
Source of infection *n* (%):	
Urine	30 (18)
Lung	81 (48.8)
Mechanical ventilation *n* (%)	36 (21.4)
Blood transfusion *n* (%)	13 (7.7)
Steroids use *n* (%)	29 (17.2)
Time for antibiotics administration	
<1 h *n* (%)	83 (50.92)
>1 h *n* (%)	80 (49.08)
AKI *n* (%)	121 (72.02)
At admission	87 (71.9)
During hospitalization	34 (28.1)
Urine output in 24 h (mL)	900 (450–1425)
Urine output (mL/kg/h)	0.70 (0.4–1.05)
APACHE II	19.67 ± 7.11
Dialysis *n* (%)	15 (8.93)
Outcome *n* (%)	
Discharge	92 (55.7)
Death	73 (44.2)

Values expressed as mean and standard deviation or median and interquartile range.

AKI: acute kidney injury, MBP: mean blood pressure, CKD: chronic kidney disease, ATN-ISS: acute tubular necrosis individual severity score, and ICU: intensive care unit.

**Table 2:** Urinary NGAL values according to presence of acute kidney injury.

	AKI *N* = 121	Non-AKI *N* = 47	*p*
uNGAL (ng/mL):			
At moment 1 (<24 h^*∗*^)	3.86 (2.6–9.54)	3.56 (0.82–5.2)	0.003
At moment 2 (24–48 h^*∗*^)	3.03 (0.65–4.33)	2.76 (2.3–7.83)	0.009
uNGAL/uCr (ng/mg):			
At moment 1 (<24 h^*∗*^)	75.08 (37–165)	53.31 (17.79–102.2)	<0.0001
At moment 2 (24–48 h^*∗*^)	77.2 (29.49–160.6)	60.29 (17.56–85.64)	0.002

Values expressed as median and interquartile range.

u: urinary; AKI: acute kidney injury; ^*∗*^after admission to emergency room.

**Table 3:** Urinary NGAL values according to patient outcome.

	Survivors *N* = 94	Nonsurvivors *N* = 74	*p*
uNGAL (ng/mL):			
At moment 1 (<24 h^*∗*^)	3.30 (1.76–6.18)	4.88 (2.19–9.51)	0.01
At moment 2 (24–48 h^*∗*^)	2.18 (0.89–6.36)	3.93 (1.89–7.19)	0.16
At moment 3 (AKI diagnosis)	6.60 (1.66–6.9)	12.42 (2.63–19.02)	0.15
uNGAL/uCr (ng/mg):			
At moment 1 (<24 h^*∗*^)	56.91 (27.55–113.92)	75.08 (40–154)	0.82
At moment 2 (24–48 h^*∗*^)	46.30 (20.81–137.49)	93.35 (57.45–115.81)	0.63
At moment 3 (AKI diagnosis)	134.41 (35.99–259.43)	263.6 (99.28–984.15)	0.056

Values expressed as median and interquartile range.

u: urinary; AKI: acute kidney injury; ^*∗*^after admission to emergency room.

**Table 4:** Urinary NGAL sensitivity and specificity in general septic patients (*n* = 168).

	AUC-ROC	*p*	Cutoff	Sensitivity	Specificity	CI (95%)
uNGAL1	0.73	0.04	3.36	0.63	0.46	(0.64–0.82)
uNGAL2	0.70	0.01	2.73	0.63	0.44	(0.55–0.85)
uNGAL/uCr1	0.77	0.04	54.8	0.70	0.38	(0.68–0.85)
uNGAL/uCr2	0.84	0.001	46.4	0.75	0.43	(0.73–0.94)

AUC-ROC: receiver operating characteristic curve; Cr: creatinine.

**Table 5:** Urinary NGAL sensitivity and specificity in septic patients without AKI at admission (*n* = 81).

	AUC-ROC	*p*	Cutoff	Sensitivity	Specificity	CI (95%)
uNGAL1	0.83	0.03	3.16	0.77	0.66	(0.64–0.81)
uNGAL2	0.81	0.01	3.83	0.75	0.78	(0.52–0.79)
uNGAL/uCr1	0.87	0.02	53.8	0.81	0.68	(0.58–0.78)
uNGAL/uCr2	0.89	0.0001	47.4	0.87	0.67	(0.64–0.71)

AUC-ROC: receiver operating characteristic curve; Cr: creatinine.

**Table 6:** Urinary NGAL sensitivity and specificity in nonsurvival septic patients.

	AUC-ROC	*p*	Cutoff	Sensitivity	Specificity	CI (95%)
uNGAL1	0.66	0.048	2.07	0.88	0.54	(0.51–0.81)
uNGAL2	0.70	0.01	1.84	0.95	0.59	(0.56–0.85)
uNGAL/uCr1	0.68	0.02	55.9	0.71	0.45	(0.54–0.83)
uNGAL/uCr2	0.81	0.001	69.6	0.71	0.48	(0.69–0.93)
